# Emotion Regulation in Participants Diagnosed With Attention Deficit Hyperactivity Disorder, Before and After an Emotion Regulation Intervention

**DOI:** 10.3389/fpsyg.2019.01092

**Published:** 2019-05-24

**Authors:** Marta Sánchez, Rocío Lavigne, Juan Fco. Romero, Eduardo Elósegui

**Affiliations:** ^1^Department of Developmental and Educational Psychology, University of Málaga, Málaga, Spain; ^2^Department of Reacher Methodology and Diagnostic Education, University of Málaga, Málaga, Spain

**Keywords:** ADHD, emotional processing, facial expression recognition, facial emotion recognition, emotional regulation problems, intervention

## Abstract

The study of Attention Deficit Hyperactivity Disorder (ADHD) addresses variables related to three core symptoms: inattention, hyperactivity, and impulsivity. However, it has been suggested that in recent years emotional difficulties and subsequent social challenges have not received sufficient attention. This study had two objectives: (1) to compare the performance of participants (age range: 8–14 years) on facial emotion recognition tasks using the Affect Recognition subtest of the Children Neuropsychological Battery II; and (2) to assess the perceptions of family members in relation to variables associated with emotional problems, difficulty in regulating emotions, and anger management using the Spanish Assessment System for Children and Adolescents. Assessments were conducted before and after applying an emotion regulation intervention designed for this study. Following the intervention, there was a significant decrease in scores associated with emotional regulation, and an improvement in the identification of affect on facial recognition tasks. The results suggest that despite ADHD children and adolescents having social and emotional deficits secondary to the core symptom triad, emotional regulation in this group can be improved by the application of socio-emotional intervention programs.

## Introduction

Attention Deficit Hyperactivity Disorder (ADHD) is one of the most common neurodevelopmental disorders in childhood and adolescence. The Diagnostic and Statistical Manual of Mental Disorders, Fifth Edition (DSM-5) (American Psychiatric Association, 2013) defines ADHD as a persistent pattern of hyperactivity, impulsivity, and inattention which is higher than that expected for the individual’s developmental level. Symptoms must be present for at least 6 months in two or more settings, they should have appeared before the age of 12 years, and they must interfere with the normal functioning of the individual. ADHD is a severe syndrome that results in a deficit in the executive control of behavior, and therefore in executive function (EF), that directly affects the academic, family, and social development of the individual (Servera, 2005; Barkley, 2006; Pérez, 2009; Lavigne and Romero, 2010).

The syndrome tends to persist into adulthood in more than half of the cases (Loro et al., 2015), hence the relevance of early diagnosis and continuous and systematic interventions addressing the patient and individuals close to them (i.e., mainly parents and teachers). Research has generally addressed the core symptom triad: inattention, hyperactivity, and impulsivity. However, there is little research on difficulties in processing emotions and self-regulation or subsequent problems in the social adaptation of ADHD individuals. Between 50 and 70% of children with ADHD experience difficulty in interacting with their classmates and typically have a low sociometric status (García et al., 2006).

These children and adolescents have an interaction pattern characterized by low levels of self-control and high levels of disruptive, aggressive, or oppositional behavior (Pardos et al., 2009; Fernández et al., 2011). In general, this behavior leads to peer rejection from the time of their first interactions, despite efforts to be accepted. Social problems increase the risk of experiencing emotional and behavioral imbalances in adolescence and adulthood. It is of note that these social problems persist despite interventions that actually decrease the impact of the core symptom triad (Mikami et al., 2014).

As early as pre-school, some parents report that their children experience difficulties with emotions, behavior, and relating to peers (Martín et al., 2017). Therefore, further research is needed on how impairments in the identification and regulation of emotions and the lack or delayed acquisition of social skills affect the individual’s ability to adapt to their settings, particularly in the context of receiving combined treatment that alleviates their core symptoms and improves their interactions in the family and school environment.

The concept of emotion refers to innate and involuntary psychophysiological mechanisms that arise as a consequence of environmental stimuli and that generate avoidance or approach reactions. Environmental stimuli are detected by the sensory cortex and processed in the limbic system by the amygdala, which is responsible for activating a physical response and for altering cognitive processing according to the emotion that is appropriate to the given stimuli. Physical reactions can be associated with the specific object that caused them, forming a propositional representation of the relationship between the emotional state and the stimulus that triggered it. Given the repeated experience of such situations and the capacity of the brain for learning, this representation would be of assistance in predicting the presence of this stimulus in a given setting and in anticipating future responses (Damasio, 2011).

Several authors have emphasized the adaptive value of emotions (e.g., Lázarus, 1991). Emotions modulate processes, such as decision making, and adapt the cognitive style of the subject to the demands of the situation, thus assisting in the performance of social functions (Gross, 1999). However, individuals need emotional management skills and strategies to regulate their states according to the demands of specific social, academic, work, or family situations. Emotional skills are a key element in appropriate psychological functioning, and various disorders are characterized by their absence or the use of dysfunctional strategies (Hervás, 2011).

Thus, adequate emotion self-regulation increases the opportunities for ADHD patients to obtain and integrate the socio-cognitive skills needed to successfully meet the demands of their environment during childhood, adolescence, and adulthood.

This aspect is particularly true in relation to adults with ADHD because the labor market has recently undergone a variety of changes with little regard for the personal needs of workers and their wellbeing (Mannino and Faraci, 2017).

There is no well-established body of models and theories that explains the origin of emotional deficits in ADHD and their associations with EF. In fact, there is little research on emotional processing and regulation in children and adults with ADHD. In general, the limited research available has taken two approaches. One line of research has investigated whether emotional impairment in ADHD is a primary or secondary deficit. Such studies have addressed the relationship between emotions, feelings, and the ability of individuals to interact appropriately in social situations. The second line has suggested that issues in emotional identification and expression are secondary to executive deficits in ADHD individuals.

Nigg and Casey (2005) suggested that impaired EF and reward systems are involved in syndromes such as ADHD, behavior disorders, addictions, or drug abuse. They proposed that these cognitive and affective mechanisms are also the basis of regulatory deficits in these disorders. Their starting point was that the neural systems involved in cognitive and affective mechanisms overlap: that is, that there is a close relationship between the neuroanatomical and neurochemical circuits responsible for psychological processes such as EF, the reward systems, emotional regulation, and motivation. These authors suggested that cognitive control could be associated with emotional responses, and that ADHD individuals may have dysfunctions in emotional and impulse control because both of these processes are affected.

Several studies have suggested that ADHD individuals have emotional reactivity traits as well as difficulties in emotional control or regulation (Banaschewski et al., 2012; Graziano et al., 2013; Petrovic and Castellanos, 2016). Emotional reactivity traits are characterized by a high degree of sensitivity to emotional stimuli due to a deficit in involuntary “bottom-up” processing, whereas emotional control or regulation directly depend on voluntary “top-down” processing that inhibits responses when needed. These deficiencies in ADHD are associated with externalizing or aggressive signs. Children with more externalizing symptoms obtained worse scores on measures of executive functioning than individuals who had more internalizing symptoms.

On the other hand, traditional theories of ADHD (Barkley, 1997; Brown, 2003; López et al., 2010) have suggested that individuals diagnosed with ADHD would have severe difficulties in regulating their emotional, motivational, and affective states, and that these aspects depend on executive system (ES) processes. Such symptoms would thus be secondary to or associated with the core symptom triad. In this sense, it is assumed that higher-level brain structures (e.g., the prefrontal cortex) exert control over lower-level structures (e.g., the limbic system).

Although “emotional arousal” is mainly regulated by the ascending reticular activating system, which connects the reticular formation and cortex, “emotional arousal” can also affect the activity of higher cognitive processes (Palmero, 1996; Ramos et al., 2009). Therefore, any disorder that affects the pathway between the prefrontal cortex and subcortical areas may give rise to executive problems in situations that require emotional control and regulation. If these emotions become very intense, the executive control system would be further affected, thus creating a vicious circle. This situation would influence the selection of the type of intervention needed. In ADHD, the ability to identify and understand emotional states in oneself or others is not affected in consequence of a primary deficit. Rather, these deficits are secondary to impaired executive functioning. The role of the ES is understood as exerting control over novel non-routine behavior aimed at a specific goal. In this disorder, impairments mainly affect emotional and impulse control, which causes irritability, aggressiveness, or low tolerance to frustration.

Similarly, the DSM-IV describes emotional impairments in ADHD as secondary to or associated with executive deficits, because they manifest as a consequence of the core symptom triad. A behavioral study (Albert et al., 2008) suggested that children with ADHD had difficulties with emotional recognition, particularly at an early age, because executive dysfunction itself (i.e., impaired frontostriatal and fronto-cerebellar circuits) hinders their ability to recognize emotional stimuli in these types of tasks. For example, difficulties in facial emotion recognition derive from their own attentional limitations when perceiving specific features and associating them with a certain emotion.

These impairments not only negatively impact emotions, but also reduce the patient’s capacity to adapt to the social setting. Generally, children and teenagers with ADHD experience difficulties other than the core symptom triad. Classmates of ADHD children complain of their interpersonal difficulties with them, and typically state that they are annoying and noisy, talk too much, interrupt conversations, do not respect game rules, or very easily become angry (García et al., 2006, 2012; Nijmeijer et al., 2008; Solanto et al., 2009; López et al., 2010; Shiels and Hawk, 2010; Graziano et al., 2013). In addition, ADHD children usually need continuous attention or supervision when assigned a particular task (e.g., following the rules in a game). They also have intrusive behavior and are not able to recognize the negative effect of this behavior on the social environment. Despite their efforts to be accepted by their peers, ADHD individuals usually obtain low scores on sociometric tests, have poor interpersonal skills, and have low social competence. Difficulties in childhood peer-relationships are often a prognostic factor of poor social development throughout life. These difficulties persist over time and tend to increase during adolescence, when disruptive and even aggressive behavior are more common.

Deficits in social and emotional skills are probably due to dysfunctions in the neurotransmitter pathways involved in emotional and motivation processes. These types of dysfunction appear to be one of the main causes of the disorder itself (Pardos et al., 2009). Changes in the understanding of the origin and characteristics of ADHD have led to changes in interventions. Intervention involves detection, assessment, prescriptive diagnosis, and treatment. However, assessment should not only be based on the information obtained by healthcare professionals during personal interviews with patients or the patient’s families. In the case of children and adolescents, assessment should include interviews with one or both parents, as well as teachers or school counselors, who can provide information about their behavior at school (Brown, 2003). This information should be complemented by questionnaires completed by the children and their parents as well as by psychometric tests of known reliability and validity.

Currently, ADHD is included in the diagnostic classifications of the International Classification of Diseases, tenth edition (ICD-10, World Health Organization [WHO], 1992) and the Diagnostic and Statistical Manual of Mental Disorders, fifth edition (DSM-5, American Psychiatric Association, 2013). Although ADHD is universally accepted as a neurodevelopmental disorder — given the scientific evidence on which it rests — assessments which exclusively address the core symptoms (i.e., inattention, hyperactivity, and impulsivity) are insufficient to diagnose the disorder. Teachers and the families of ADHD children are often concerned about problems other than the core symptom triad, such as disobedience, emotional lability, difficulty in making friends, family relationships, or learning difficulties (Vaquerizo, 2008). Thus, the presence of deficits or impairments in the daily life of ADHD children implies the need to pay particular attention to social and emotional variables. These variables should be assessed and, if needed, quantified through specific tests and taken into account during assessment and diagnosis.

The subsequent intervention phase involves treatment procedures. These have proliferated in recent years, and some of them are based on excessive claims that lack scientific rigor. At present, such procedures can be classified into pharmacological, behavioral, cognitive-behavioral, and psychoeducational treatments. These treatments are sometimes administered alone, but typically two or more treatments are used in combination. The effectiveness of each type of treatment varies depending on the aspects of the disorder being addressed. However, there is a consensus among researchers and professional therapists that the most effective approach is the use of combined pharmacological and psychoeducational treatment. Some researchers have suggested that this combination includes more variables, while professional therapists have suggested that it can be applied to a variety of settings (e.g., clinical practice, home, school, and social situations) (Tuchman, 2001; Loro et al., 2009; Pérez, 2009; Lavigne and Romero, 2010; Montañés et al., 2010; Mulas et al., 2012).

Combined pharmacological and psychoeducational treatment significantly reduces the core symptoms of the disorder and improves self-control, thus reducing problems associated with learning and with adapting to the environment (Miranda et al., 2000; Valdizán, 2004; Escobar et al., 2005). This line of treatment includes parent training and sessions for the whole family (Valls et al., 2015). They are taught behavior modification techniques and cognitive and problem-solving techniques. The aim is to reduce oppositional behavior in children and adolescents and to improve communication between parents and children. Some studies (e.g., Hawes et al., 2011) have suggested that the use of effective discipline practices by parents of ADHD children improves self-regulation capacity at an early age, whereas inconsistent discipline patterns contribute to the aggravation of core symptoms in mid-childhood. Inconsistent discipline is also associated with high levels of behavioral problems, defiant opposition, and peer rejection (Miranda et al., 2008). However, emotional processing problems, and thus social interaction problems, continue to manifest even after the application of multidisciplinary and multicomponent treatment (Miranda et al., 2000; Valdizán, 2004; Escobar et al., 2005).

The present study included a sample of 64 children and adolescents diagnosed with ADHD (age range: 8–14 years). The study had two objectives: (1) to compare their performance on facial emotion recognition tasks before and after an intervention specifically designed to improve affect recognition (AR) and emotional regulation; and (2) to compare the perceptions of 110 participating parents in relation to variables associated with emotional problems, difficulty in regulating emotions, and anger management before and after training with their children.

## Materials and Methods

### Participants and Method

The study was conducted during the 2015–2016 school year by the HUM-347 research group from the Department of Developmental and Educational Psychology at the University of Malaga (Spain) and the team from the Centro de Diagnóstico y Tratamiento de TDAH [ADHD Diagnosis and Treatment Center (ADHD-DTC)] in Malaga. The ADHD-DTC is a private therapeutic center managed by a multidisciplinary team that includes a neuropediatrician, two psychologists, and three educational psychologists. The center mainly treats individuals diagnosed with ADHD, but it also treats individuals with learning difficulties and behavior disorders. All clients are drawn from Andalusia, Ceuta, and Melilla (Spain).

The study design was quasi-experimental. Partially repeated measures (A-B-A) were obtained before and after conducting an intervention that was specifically designed for this study. In the pretest phase, clients made their first contact with the TDAH-CDT and the research project was described to them. Recruitment was conducted by e-mail or telephone.

The final sample was selected according to the following inclusion criteria:

○ Having a diagnosis of combined-type ADHD, predominantly inattentive ADHD, or predominantly hyperactive/impulsive ADHD. Symptoms must have been present for more than 6 months and in at least two different settings.
○ Clear evidence of a clinically significant deterioration in social and/or emotional activity.
○ No signs of schizophrenia, psychotic disorder, or any other mental disorder. No signs of intellectual disability and no sensory or motor deficits.
○ An IQ score equal to or more than 80 on the Wechsler Intelligence Scale for Children-Revised (WISC-R) or on the Wechsler Intelligence Scale for Children-IV (WISC-IV).
○ Being between 7 and 14 years old.
○ No previous or current treatment specific to the social and emotional domains.

The initial sample comprised 130 children and adolescents, of which 64 (52 males and 12 females) met the inclusion criteria. The study included 110 parents, who signed the informed consent form and were briefed on the different phases and characteristics of the study. The ADHD participants and their families were tested in the pretest and posttest phases using two instruments (see below for details).

We designed an emotion regulation intervention (ERI) that included AR and regulation tasks. Particular attention was given to negative primary emotions, such as rage, sadness, and fear. This intervention mainly addressed the acquisition and development of emotional identification, expression, and regulation techniques. Some activities were based on the recognition of primary emotions through faces, the analysis of social situations with emotional content, and the acquisition of cognitive strategies to express emotions in an appropriate manner while taking the setting into account. The intervention also included strategies to control and regulate negative emotions (mainly rage, sadness, and fear). After each session, parents received advice on how to achieve the goals pursued in each session, and were given guidelines on how to help their children apply what they had learned to other settings.

The ERI was based on our review of Barkley’s (2006) hybrid model of self-regulation and EFs, Lavigne and Romero’s (2009) model of deficits in the ES that controls EFs, Sonuga’s (2005) delay aversion and dual pathway models, Nigg and Casey’s (2011) integrative theory of ADHD, and Damasio’s (2011) somatic markers hypothesis. This approach allowed us to deepen our understanding of the limitations ADHD patients experience in social and emotional settings.

In order to develop the overall design of the intervention, we also reviewed classic pyschoeducational intervention programs for normotypical individuals and ADHD patients (Orjales and Polaino, 2002; Roca, 2003; Cobo and Galindo, 2007; Monjas, 2007; Iribarren et al., 2010; Méndez et al., 2012).

Different activities were designed for two age ranges (8–9 years and 10–14 years). Each activity had the same goal, but the presentation format and the level of difficulty varied according to age range. The intervention consisted of 10 sessions. The first and last sessions were used to administer the pretest and posttest assessments, and thus evaluate the efficacy of treatment. The intervention comprised cognitive, behavioral, and emotional dimensions. Each session comprised 60 min of working with the children, and at least 15 to 20 min of working with their parents. The children were taught specific strategies and skills, and how and when to use them. Information was provided to parents on what the children had learned in each session, guidelines were established, and questions and concerns were solved. Finally, the parents were given advice on how to reinforce at home the skills the children had learnt.

The intervention was administered by dividing the ADHD group into 15 groups of three to five participants. The groups were formed according to age range and availability (i.e., morning or afternoon). There were 6 groups for ages 8 to 9 years (total: 30 participants), 4 groups for ages 10 to 11 years (total: 14 participants), and 5 groups for ages 12 to 14 years (total: 20 participants).

### Instruments

Pretest and posttest data were collected using the following instruments:

• The AR subtest of the NEPSY-II Neuropsychological Battery (social perception domain) (Korkman et al., 2014). This instrument is used to collect information on different cognitive domains. The AR subtest is designed to assess the ability to recognize primary emotions (sadness, fear, disgust, and anger). The participants perform four tasks in which they are show photographs of children’s faces. In one of the tasks, the child states whether or not two of the photographs show faces with the same affect. A correct match receives a score of 1 and 0 otherwise. Direct scores can be ranked into percentiles such that the participant’s performance can be compared to that of the general population. The final AR score is the sum of the scores obtained on each of the test items. The lowest scores on this variable indicate that the participants have difficulties performing facial AR tasks.

The Emotional Problems, Emotional Regulation Problems, and Anger Control Problems subtests of the Spanish Assessment System for Children and Adolescents (SENA) (Fernández et al., 2015). This scale is designed to collect information on behavioral and emotional problems using self-report questionnaires. There is one format for parents and another for children and teenagers (i.e., 8–12 years and 12–18 years, respectively). Items in the parent’s and teenager’s questionnaires are scored on a 6-point Likert scale (i.e., never, almost never, rarely, sometimes, frequently, almost always). For reasons of simplicity, the children’s questionnaire uses a 3-point Likert scale (yes, no, and sometimes). The final scores were processed for analysis using the “TEA Corrige” online platform^1^. Direct scores and T-scores were used to obtain a profile for each subject and family.

We chose the general sample (male and female) from the different normative samples available to evaluate the results of the participants. High scores on these variables indicate that the participants have emotional, emotion regulation, and anger management issues.

## Results

All data were analyzed using the SPSS Statistical software package, version 23. The efficacy of the ERI was analyzed by comparing the pretest and posttest mean scores of the psychometric tests and the self-report questionnaires using a paired sample *t*-test.

**FIGURE 1:**
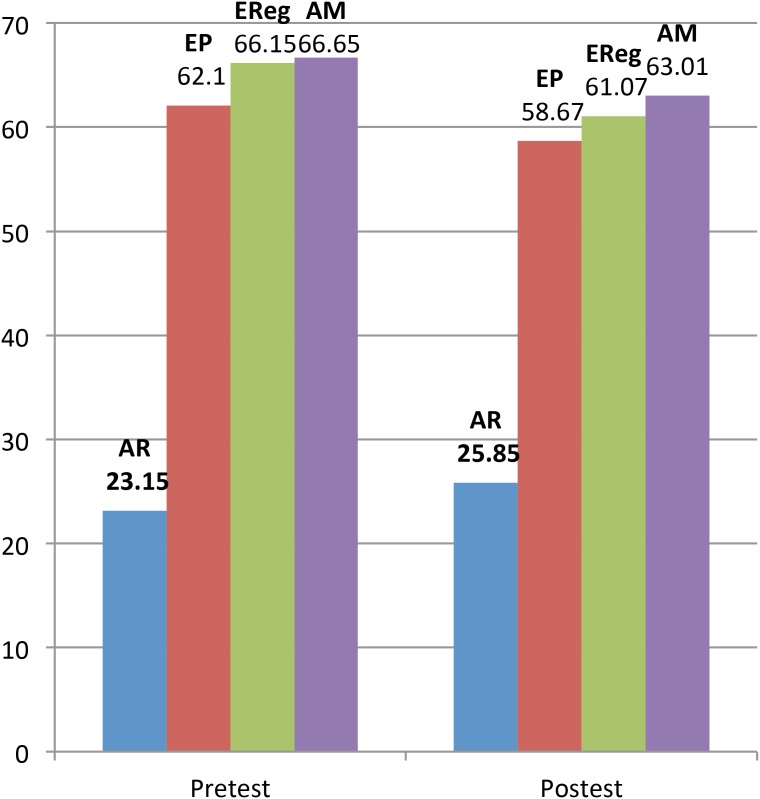
Objectives 1 and 2. Comparison of results pre- and post-ERI. AR, affect recognition; EP, emotional problems; EReg, emotional regulation; AM, anger management.

Table 1 shows the overall ERI pre- and post-test results. Tables 2–5 show the ERI pre- and post-test results, respectively, on facial AR tasks, emotional problems, emotional regulation problems, and anger management problems. Finally, Figure 1 shows the ERI pre- and post-test results on all variables.

**Table 1:** Objective 1 and Objective 2. Comparison of results pre- and post-ERI.

Pretest and posttest results for O1 and O2	O1: AR	O2: EP	O2: EReg	O2: AM
Pretest	23.15	62.10	66.15	66.56
Posttest	25.85	58.67	61.07	63.01
Significance level	0.00	0.01	0.00	0.02


*O1, objective 1; O2, objective 2; AR, affect recognition; EP, emotional problems; EReg, emotional regulation; AM, anger management.*

**Table 2:** Objective 1. NEPSY-II: results pre- and post-ERI.

Objective 1. NEPSY-II	AR
Pretest	23.15
Posttest	25.85
Mean difference	–2.70
T	–3.83
DF	63
Significance level	0.00


*NEPSY-II, neuropsychological battery; AR, affect recognition; DF, degrees of freedom.*

In relation to AR, significant differences were found between the means of the pretest phase (23.15) and posttest phase (25.85) [*t*(63) = -3.83; *P* = 0.00; see Table 2]. Significant differences were found between the results of the pretest and posttest phases [*t*(63) = 2.63; *P* = 0.01] in emotional problems. The parents reported that their children had fewer emotional problems after the intervention (see Table 3).

**Table 3:** Objective 2. SENA – Emotional problems: results pre- and post-ERI.

Objective 2. SENA	EP
Pretest	62.10
Posttest	58.67
Mean difference	3.43
T	2.63
DF	63
Significance level	0.01


*SENA, Spanish assessment system for children and adolescents; EP, emotional problems; DF, degrees of freedom.*

Significant differences were found between the pretest mean (66.15) and posttest mean (61.07) in emotional regulation [*t*(63) = 4.11 and *P* = 0.00]. The parents reported that their children had less difficulty in emotional regulation after the intervention (see Table 4) Similarly, significant differences were also found between the pretest mean (66.56) and posttest mean (63.01) in anger management [*t*(63) = 2.36 and *P* = 0.02; see Table 5]. These results are discussed in detail in the next section.

**Table 4:** Objective 2. SENA – Emotional regulation: results pre- and post-ERI.

Objective 2. SENA	EReg
Pretest	66.15
Posttest	61.07
Mean difference	5.07
T	4.11
DF	63
Significance level	0.00


*SENA, Spanish assessment system for children and adolescents; EReg, emotional regulation; DF, degrees of freedom.*

**Table 5:** Objective 2. SENA – Anger Management: results pre- and post-ERI.

Objective 2. SENA	AM
Pretest	66.56
Posttest	63.01
Mean difference	3.54
T	2.36
DF	63
Significance level	0.02


*SENA, Spanish assessment system for children and adolescents; AM, anger management; DF, degrees of freedom.*

The results show that after the ERI there were significant improvements in the children’s performance on AR tasks, and that after training the parents reported a significant decrease in their children’s emotional problems, better regulation of negative emotions, and increased anger control. However, a literature search showed that there is little research on socio-emotional deficits in ADHD individuals and on the different options for diagnosis and treatment, which was confirmed by Albert et al. (2008). Therefore, it is difficult to compare our results to those of previous studies. Some descriptive and behavioral studies (Wender et al., 1983, 1993; Shapiro et al., 1993; Reimherr et al., 2005; Uekermann et al., 2010) have documented the socio-emotional problems of ADHD patients. However, the empirical literature is limited (Miranda et al., 2000; Montiel et al., 2002; Arco et al., 2004; Lavigne and Romero, 2010).

## Discussion

### Objective 1: To Test ADHD Participants on Facial Recognition Tasks Using the Affect Recognition Subtest of the NEPSY-II

In relation to AR, significant differences were found between the means of the pretest phase (23.15) and posttest phase (25.85) [*t*(63) = -3.83; *P* = 0.00].

Some studies have also suggested that identifying the emotions of others is more difficult for ADHD individuals than for normotypical individuals (Shapiro et al., 1993; Uekermann et al., 2010). Both these studies reported a deficit in facial emotion recognition. We found a similar effect, which was particularly evident in the younger ADHD participants (8–10 years). Rodrigo et al. (2017) conducted a systematic review of the scientific literature on studies that used AR in facial expression tasks to establish the presence or absence of emotional deficits in ADHD individuals. They found that 70% of the studies reviewed suggested that there were significant differences in AR between ADHD children and normotypical children.

Some studies have found that the recognition of negative emotions, such as fear or anger, is more difficult for ADHD individuals than for normotypical individuals. However, further research is needed to derive definitive conclusions given that some studies have been limited by comorbidities in the participants or by the absence of a control group. Uekermann et al. (2010) described difficulties not only in the recognition of facial affect, but also in the perception of emotional states and prosody, particularly in younger individuals. However, other studies have found fewer differences in facial emotion recognition between ADHD children and normotypical children (Singh et al., 1998). Unfortunately, we were unable to find any study that had an objective similar to our first objective, used an A-B-A design, and applied the same instruments. Therefore, our results on the efficacy of ERI cannot be compared to those of other interventions.

### Objective 2: To Assess the Perceptions of Family Members in Relation to Emotional Problems, Emotional Regulation, and Anger Management in ADHD Children Using the SENA Family Questionnaires

Significant differences were found between the results of the pretest and posttest phases [*t*(63) = 2.63; *P* = 0.01] in emotional problems. The parents reported that their children had fewer emotional problems after the intervention.

Several authors (Wender et al., 1993; Reimherr et al., 2005) have reported affect problems such as emotional lability, excessive emotional reactivity, and irritability in adults diagnosed with ADHD whose core ADHD symptoms had responded well to psychostimulant treatment (i.e., methylphenidate or atomoxetine). These findings, together with the improvements reported in this study, justify the need to investigate the specific problems ADHD individuals experience in expressing and modulating negative emotional states. Childhood interventions could help to decrease the risk of such problems becoming chronic. Given that the disorder continues into adulthood, and may become acute, early diagnosis and continuous interventions are essential.

Significant differences were found between the pretest mean (66.15) and posttest mean (61.07) in emotional regulation [*t*(63) = 4.11 and *P* = 0.00]. The parents reported that their children had less difficulty in emotional regulation after the intervention. Similarly, significant differences were also found between the pretest mean (66.56) and posttest mean (63.01) in anger management [*t*(63) = 2.36 and *P* = 0.02].

Other studies (Buhrmester et al., 1992; Kitchens et al., 1999; Maedgen and Carlson, 2000; Jensen and Rosén, 2004; Wehmeier et al., 2010) have highlighted the difficulties of ADHD individuals in trying to control negative emotions, such as anger, fear, and sadness, due to their having more extreme reactions and higher levels of aggressiveness than those found in the general population. Although we observed improvements in emotional regulation after the intervention, Walcott and Landau (2004) reported that ADHD individuals had difficulty in regulating their emotions even after receiving specific advice on how to do so.

Our results are consistent with those of other studies (Miranda et al., 2000; Montiel et al., 2002; Arco et al., 2004) that had a similar design to that of our study, although the data collection instruments used were different. These studies included socio-emotional variables in their interventions, and parents and teachers participated in the intervention. These studies found statistically significant improvements in the study variables after the different groups underwent specifically designed interventions. Miranda et al. (2000) found significant improvements in self-control after the intervention. Herrero et al. (2010) analyzed the effects of a psychosocial intervention on the academic, emotional, and social adaptation of 27 children diagnosed with ADHD. They found significant improvements in their emotional adaptation and a decrease in anxiety problems.

Hughes et al. (2000) studied preschool children who had symptoms compatible with ADHD. These authors suggested that manifestations of anger and dissocial behavior in these children were associated with poor performance in inhibition and planning tasks. In the same line, Graziano et al. (2013) assessed the co-occurrence of internalizing and externalizing symptoms in a sample of 74 ADHD children and adolescents (age range: 6–17 years). The authors found that the participants who had poor performance on EF tasks had emotional reactivity problems and externalizing/aggressive symptoms, whereas those who had higher levels of internalizing symptoms had a decreased probability of executive deficits. Banaschewski et al. (2012) analyzed the relationship between ADHD and emotional lability in order to clarify whether lability depended on cognitive or motivational dysfunctions, and whether this association was mediated by the core symptom triad. The sample comprised 424 children and adolescents (age range: 6–18 years) who had been diagnosed with ADHD using a neuropsychological battery. The control group comprised 564 participants. It was found that the neuropsychological imbalances involved in ADHD were predictors of emotional lability, which would suggest that the neuropsychological functions and their neurological substrates were functionally involved in emotional lability.

Banaschewski and other authors have suggested that the severity of ADHD symptoms may statistically mediate the relationship between neuropsychological dysfunctions and symptoms of emotional lability (Sobansky et al., 2010; Banaschewski et al., 2012). However, the literature search failed to find any experimental study with which to compare our results.

Research has suggested that specific interventions on cognitive variables, significantly reduces symptoms and improves the interactions between children and adolescents and their environment, particularly when the intervention includes combined treatment and family participation (Loro et al., 2009; Lavigne and Romero, 2010; Montañés et al., 2010; Mulas et al., 2012). The results support the view that interventions, such as ERI, that address emotional and social variables could complement ADHD treatment and increase its efficacy. However, to date, these kinds of interventions have received little attention. Therefore, it would be of interest to conduct further studies using an A-B-A design and apply interventions that include cognitive, social, and emotional variables. It would thus be possible to investigate potential correlations between improvements on cognitive process and function tasks and better performance on emotional and social variable tasks.

## Conclusion

In recent decades, ADHD has become one of the neurodevelopmental disorders receiving more attention from researchers and professionals working in psychology, education, and medicine. Advances in this area have led to the development of new explanatory neuropsychological models of the disorder, consensus guidelines for its detection and diagnosis, and pharmacological and psychoeducational treatment strategies increasingly adapted to the needs of ADHD individuals.

Within this setting, genetic factors that modulate biochemical neurotransmission processes that interfere with the correct development and functioning of the ES have been found to be relevant. However, research suggests that most ADHD individuals have emotional control and regulation problems, leading to difficulties in social adaptation. These problems increase with age and persist despite the application of interventions to reduce the core symptom triad.

The scientific literature is replete with descriptions of these difficulties, drawing a portrait of individuals who do not understand their own emotions or who are unable to control and regulate them, do not understand the emotions of other people, and even have problems recognizing them correctly. As a consequence, such individuals exhibit maladaptive social behavior (e.g., breaking norms, disruptive behavior, aggression, and lack of conflict-solving skills).

One of the motivations for this study was precisely the lack of attention given to the study and creation of treatment programs and strategies to modify inappropriate social interaction patterns, which derive from impaired emotional, cognitive, and socio-communicative processing. These impairments lead to peer rejection and increase the risk of experiencing emotional imbalances in childhood, adolescence, and adult life.

The results show that the identification of emotional states in others and the application of negative emotional control strategies, as well as emotional regulation problems, can be improved in ADHD individuals. These improvements can be achieved if it is assumed that one of the primordial functions of the ES is the control and regulation of emotions, and that this system plays a leading role in the development of feelings and the management of social skills. Treatment should be applied using combined programs in which participants are given strategies and skills to decrease the impact of ADHD deficits. If possible, interventions should be implemented in young ADHD individuals to prevent disruptive behavior becoming habitual and the development of negative thoughts about themselves, which may lead to feelings of low self-esteem.

In summary, the difficulties that ADHD individuals experience when they attempt to identify, express, and regulate emotions are due to the delayed development of emotional processing skills caused by the executive deficits that characterize the disorder. These difficulties could be improved by the application of an early multidimensional intervention that includes emotional variables and is combined with pharmacological and psychoeducational treatment.

Future lines of research could extend the focus of the present study and address some of the limitations of our study. Firstly, it would be of interest to compare the performance of ADHD participants and a control group of normotypical participants. Our study addressed the improvement of deficits related to ES functioning in ADHD individuals. However, it would be of interest to compare our results with those of a non-ADHD population of the same age and sex. Furthermore, we would compare variables as subjective perception of time, well-being and happiness (Mannino and Caronia, 2017; Mannino et al., 2017). Such results would serve as a reference to compare the pre- and post-intervention performance of ADHD individuals on socio-emotional tasks to that of the general population. This approach would help in the development and improvement of assessment and intervention strategies, increase the understanding of deficits in the ADHD population, and open the way to new lines of research.

The inclusion and exclusion criteria used in this study were established before the sample was selected in order to control for as many confounding variables as possible (e.g., comorbidities or associated disorders). In subsequent studies, it would be of interest to begin the selection process at the assessment stage, leading to better control of the confounding variables. It could also be of interest to include groups with comorbid disorders (e.g., behavioral disorders or autistic spectrum disorders), such that their performance could be compared and phenotypes established based on diagnostic labels (Cobos et al., 2012; Granieri et al., 2017; Lo Coco et al., 2018; Sideli et al., 2018).

In summary, the main findings of this study show that emotional difficulties in individuals diagnosed with ADHD, and their resulting social challenges, are relevant issues that should receive the same level of attention as the three primary symptoms of this disorder. The present study had two aims: (1) to compare the performance of ADHD patients on facial AR tasks; and (2) to assess the perceptions of family members in relation to variables associated with emotional problems, difficulty in regulating emotions, and anger management. Assessments were conducted before and after applying an ERI specifically designed to identify and regulate emotions. Our results suggest that although children and adolescents with ADHD show social and emotional deficits secondary to the core symptom triad, emotional regulation in this group can be improved by socio-emotional intervention programs that complement classic treatment packages.

## Ethics Statement

This study was carried out in accordance with the recommendations of the Faculty of Educational Sciences and the Regulations of the Ethical Committee of Experimentation of the University of Malaga. In addition, it complies with the requirements of the Organic Law on Data Protection 3/2018, in force in Spain. Ethics approval was not required as per the University of Malaga’s guidelines and national regulations. Written informed consent was obtained from the parents of all participants.

## Author Contributions

MS collected and processed the experimental data, performed the analysis, drafted the manuscript, designed the figures, and wrote the manuscript with support from RL and JR. RL and JR conceived the presented idea, involved in the planning, and supervised the work. EE worked out almost all of the technical details, and performed the numerical calculations for the suggested experiment.

## Conflict of Interest Statement

The authors declare that the research was conducted in the absence of any commercial or financial relationships that could be construed as a potential conflict of interest.

## Footnotes

1 http://web.teaediciones.com/on-line.aspx
